# Dynamic Bidirectional Associations Between Global Positioning System Mobility and Ecological Momentary Assessment of Mood Symptoms in Mood Disorders: Prospective Cohort Study

**DOI:** 10.2196/55635

**Published:** 2024-12-06

**Authors:** Ting-Yi Lee, Ching-Hsuan Chen, I-Ming Chen, Hsi-Chung Chen, Chih-Min Liu, Shu-I Wu, Chuhsing Kate Hsiao, Po-Hsiu Kuo

**Affiliations:** 1 Department of Public Health and Institute of Epidemiology and Preventive Medicine College of Public Health National Taiwan University Taipei Taiwan; 2 Department of Obstetrics and Gynecology Taipei City Hospital Heping Fuyou Branch Taipei Taiwan; 3 Department of Psychiatry National Taiwan University Hospital Taipei Taiwan; 4 Department of Psychiatry Center of Sleep Disorders National Taiwan University Hospital Taipei Taiwan; 5 Department of Psychiatry Mackay Memorial Hospital Taipei Taiwan; 6 Department of Medicine MacKay Medical College New Taipei City Taiwan; 7 Institute of Health Data Analytics and Statistics College of Public Health National Taiwan University Taipei Taiwan; 8 Psychiatric Research Center Wan Fang Hospital Taipei Taiwan

**Keywords:** ecological momentary assessment, digital phenotyping, GPS mobility, bipolar disorder, major depressive disorder, GPS, global positioning system, mood disorders, assessment, depression, anxiety, digital phenotype, smartphone app, technology, behavioral changes, patient, monitoring

## Abstract

**Background:**

Although significant research has explored the digital phenotype in mood disorders, the time-lagged and bidirectional relationship between mood and global positioning system (GPS) mobility remains relatively unexplored. Leveraging the widespread use of smartphones, we examined correlations between mood and behavioral changes, which could inform future scalable interventions and personalized mental health monitoring.

**Objective:**

This study aims to investigate the bidirectional time lag relationships between passive GPS data and active ecological momentary assessment (EMA) data collected via smartphone app technology.

**Methods:**

Between March 2020 and May 2022, we recruited 45 participants (mean age 42.3 years, SD 12.1 years) who were followed up for 6 months: 35 individuals diagnosed with mood disorders referred by psychiatrists and 10 healthy control participants. This resulted in a total of 5248 person-days of data. Over 6 months, we collected 2 types of smartphone data: passive data on movement patterns with nearly 100,000 GPS data points per individual and active data through EMA capturing daily mood levels, including fatigue, irritability, depressed, and manic mood. Our study is limited to Android users due to operating system constraints.

**Results:**

Our findings revealed a significant negative correlation between normalized entropy (*r*=–0.353; *P*=.04) and weekly depressed mood as well as between location variance (*r*=–0.364; *P*=.03) and depressed mood. In participants with mood disorders, we observed bidirectional time-lagged associations. Specifically, changes in homestay were positively associated with fatigue (β=0.256; *P*=.03), depressed mood (β=0.235; *P*=.01), and irritability (β=0.149; *P*=.03). A decrease in location variance was significantly associated with higher depressed mood the following day (β=–0.015; *P*=.009). Conversely, an increase in depressed mood was significantly associated with reduced location variance the next day (β=–0.869; *P*<.001). These findings suggest a dynamic interplay between mood symptoms and mobility patterns.

**Conclusions:**

This study demonstrates the potential of utilizing active EMA data to assess mood levels and passive GPS data to analyze mobility behaviors, with implications for managing disease progression in patients. Monitoring location variance and homestay can provide valuable insights into this process. The daily use of smartphones has proven to be a convenient method for monitoring patients’ conditions. Interventions should prioritize promoting physical movement while discouraging prolonged periods of staying at home.

## Introduction

Patients with major depressive disorder (MDD) and bipolar disorder (BP) frequently experience symptoms during abnormal mood swings. These mood symptoms not only affect an individual’s overall functioning and quality of life but also influence their behavior and activity patterns. Traditional methods of measuring mood were limited to subjective self-reporting, which lacked the frequency, convenience, and long-term detection required for necessary assessment. With the advent of digital phenotyping [1], collecting both active and passive data is now possible in a more timely and efficient manner. Digital phenotyping represents an unobtrusive and nonintrusive surveillance approach, harnessing the growing availability of health-related data to enhance our understanding of disease-related status [2]. Currently, mental disorder assessment heavily relies on subjective measures such as paper-based questionnaires used in clinical diagnosis. Additionally, doctors frequently have only a few minutes to assess mood disorders, making a thorough evaluation of symptoms and behaviors challenging within a limited time frame.

A smartphone app has been developed to gather digital phenotypic data from users, enabling the use of participants’ medical data as a foundation for developing systems for long-term tracking and personalized health care [3]. The usage and ownership of smartphones far surpass that of wearable devices, while wearable devices have gained some popularity [4]. Smartphones not only allow for passive data collection but also allow active data collection through techniques such as ecological momentary assessment (EMA) [5]. EMA involves real-time, active self-reporting with smartphones or actigraphy, which captures participants’ current behavior and experiences in their natural environment [6]. Individuals can fill out surveys multiple times per day through EMA, enabling a better understanding of symptom variability over time [6]. EMA on smartphones aims to minimize recall bias, maximize generalizability, and study the effects of high-frequency surveys on behavior in real-world settings [5].

Recent studies have introduced novel approaches to measure behavior and activity by using global positioning system (GPS) geographic location data as reliable indicators of distance travelled or daily movement, which can be associated with mood fluctuations [7]. Symptoms associated with MDD include energy level changes and feelings of fatigue [8], while symptoms of BP often involve irritability. Several studies have revealed a correlation between reduced daily movement activity and increased depressive symptoms at a 2-week follow-up [9,10]. Additionally, studies have examined the predictive value of changes in GPS features related to locations and transitions in determining variations in depression symptom severity [11]. Conversely, emotions have been found to affect subsequent mobility behavior. Baseline measurements of depressive symptom severity have been shown to affect GPS-based indices over a 2-week follow-up period, including circadian movement, location variance, and normalized entropy, which measures the distribution of a participant’s time across different location clusters [12,13]. Additionally, increased homestay is associated with greater depressive symptom severity [14]. These studies often investigate the relationships between mood and behavioral patterns from a time lag perspective.

The infrequent assessment of mood, which is typically conducted every 2 or 3 weeks [4,15-17], limits our ability to capture the dynamic changes in mood experienced by individuals. Tracking patients’ behavior and mood in a timelier manner is essential to effectively monitor these fluctuations. Assessing emotions at baseline and again after 2 weeks is insufficient to capture individual changes over time. Therefore, this study aims to monitor daily mood variations, focusing on day-to-day changes rather than long-term shifts from baseline to 2 weeks later. This is achieved by implementing a daily self-reporting approach over 6 months, capturing participants’ daily experiences. The aims of this study are to (1) perform the GPS feature selection by integrating EMA data across all participants and (2) explore the temporal bidirectional relationship between GPS features and EMA data over 6 months in individuals with mood disorders, with a particular emphasis on time lag effects.

## Methods

### Recruitment

We conducted a prospective study to continuously collect passive smartphone GPS data and daily EMA data on mood-related symptoms over a 6-month period. Participants were enrolled from psychiatric clinics in 3 major hospitals in Taipei between March 2020 and June 2022. Notably, the peak of the COVID-19 outbreak in Taiwan began in May 2022. Despite the global panic induced by the COVID-19 pandemic, Taiwan’s prompt response effectively mitigated local transmission rates [18]. Consequently, the impact of actual infections and related preventive measures on GPS data collection was significant only after May 2022 and not during the initial recruitment phase. This study targets outpatients aged 20-65 years who met the Diagnostic and Statistical Manual of Mental Disorders criteria for MDD or BP. All participants were diagnosed by psychiatrists and consented to formal interviews conducted by our team interviewers. Patients who are night-shift workers, with substance-induced mood disorders, intellectual developmental disorder, or schizophrenia were excluded. Healthy controls were recruited from the community near the recruitment hospitals in Taipei, specifically targeting non–night shift workers and individuals with no history of psychiatric disorders. Initially, 95 participants were eligible for recruitment. However, due to factors such as inability to establish contact or the experience of panic attacks, only 45 participants were successfully enrolled who completed the questionnaires. Consequently, our study focused on these 45 participants, tracking their daily moods over a 6-month period. This extended monitoring allowed us to observe variations in the mood states of the participants over time. In analyzing the correlation between mood levels and mobility features derived from GPS data, we included data from healthy controls to identify the key variables for subsequent time lag analysis. The time lag analysis itself was confined to data from participants diagnosed with MDD and BP.

### Ethics Approval

All participants provided written informed consent, and this study received approval by the institutional review boards of National Taiwan University Hospital (Approval number: 201811087RINA).

### Measurement of Symptom Severity

We investigated whether the patients were in the acute stage at baseline by using the Young Mania Rating Scale (YMRS) and Hamilton Depression Rating Scale (HAMD) scores for measurement to understand the disease severity. In this study, depressive symptoms and their severity were evaluated using HAMD [19]. The HAMD scores are interpreted as follows: 0-7 indicates a normal range, 8-16 suggests mild depression, 17-23 corresponds to moderate depression, and a score over 24 signifies severe depression. For assessing manic symptoms, YMRS [20] was employed. In YMRS, scores of 0-11 indicate remission, 12-15 suggest mild symptoms, 16-20 are indicative of moderate symptoms, and 21-60 represent severe symptoms. The cutoff value of <16 on YMRS denotes a nonacute stage of manic symptoms. The Beck Depression Inventory-II [21] measures the severity of symptoms, assessing the current depressive severity over the past 2 weeks. The scale classifies depression severity as follows: 0-13 indicates minimal, 14-19 indicates mild, 20-28 indicates moderate, and 29-63 indicates severe depression. All patients in our study were not in the acute stage of their conditions.

### GPS Features and EMA Mood Collection

**Figure 1:**
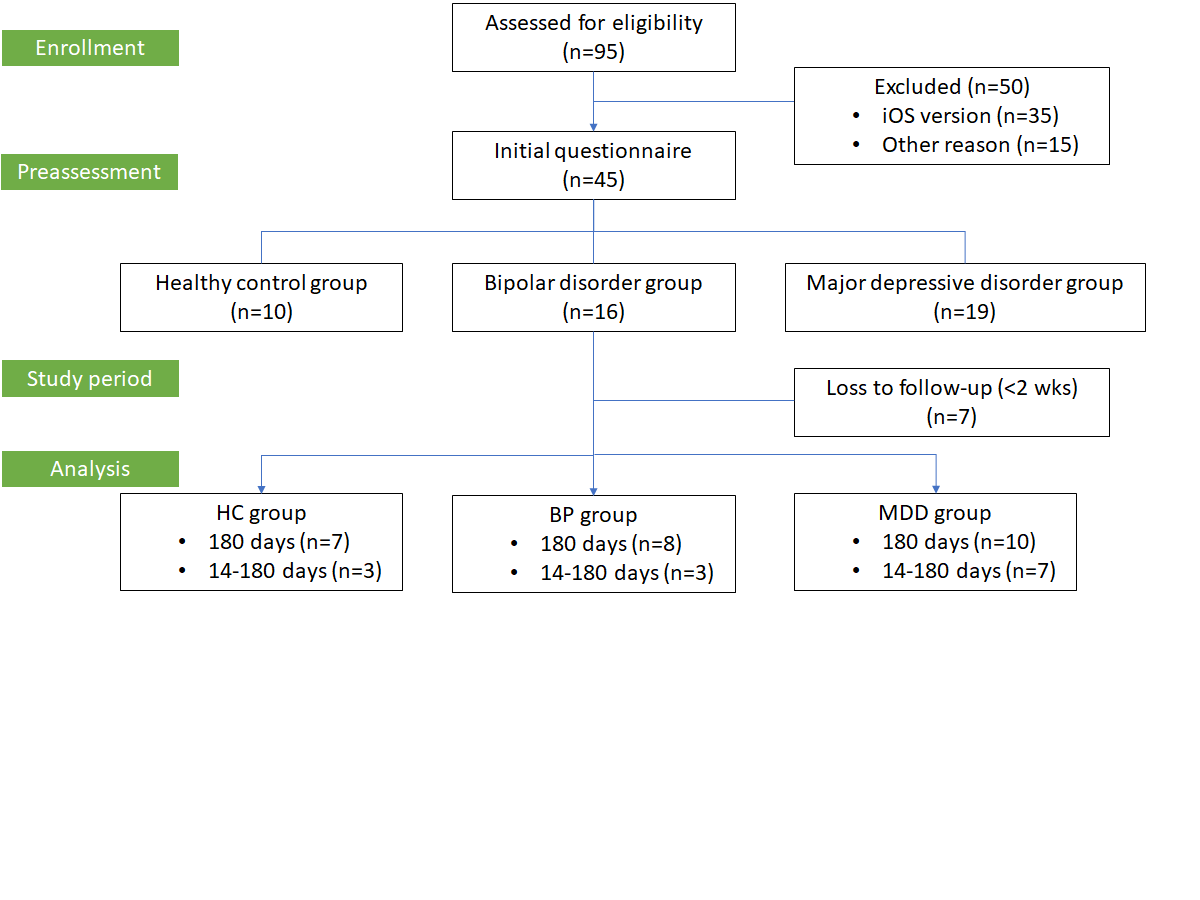
Flowchart of the 6-month study period. BP: bipolar disorder; HC: healthy control; MDD: major depressive disorder.

We collected GPS and EMA data by using the Beiwe platform developed by the Harvard research team Onnela Lab (Multimedia Appendix 1). It extracts biomedical and clinical insights from smartphone data by using research platforms, mobile apps, databases, and data analysis tools [2]. We collected data from participants who started to use the Beiwe app on March 16, 2020. The smartphone app is applied to Android and iOS systems as part of the Beiwe research platform [22]. Therefore, we could collect raw data from individual-level participants at every moment in actual real-world experiences everywhere. However, Beiwe was presented in English version; so, we worked with the Beiwe research team to develop a traditional Chinese version suitable for our study. Two types are currently available: Android and iOS versions. The Android version was easier to launch and has begun to enroll participants. However, problems remain with the data collection of the iOS system in the Chinese version; thus, our study can only extract data from the Android version. Therefore, out of the 95 eligible participants, 35 participants were excluded who were using the iOS version (Figure 1).

The GPS data includes a timestamp, Universal Time Coordinated, longitude, latitude, altitude, and accuracy. The time recorded by the Beiwe platform is Universal Time Coordinated. Taiwan time is 8 hours ahead of Universal Time Coordinated; thus, it was converted to Taiwan time. We collected GPS data generated by smartphone sensors 1000 times a day for 1 participant and realized the path of movement [22]. The GPS could not be sampled continuously because this would drain the smartphone’s battery within a few hours; so, the app operated the GPS sensor at a prespecified frequency and regular duration intervals [2]. The Beiwe platform uploads an Excel file every hour, which is set as a rest interval of every 10 minutes to record 1 minute of data. Ideally, 1 data would record per second, but sometimes, the number of uploads would vary depending on the smartphone type [16]. The GPS data collected on the phone were encrypted at the moment of collection, and the recorded GPS data were not in the original latitude and longitude format. Additionally, the GPS data could not be accessed on the phone itself, and encryption measures protected the transmission process. Only when the data reached the researchers could it be decrypted to obtain the original GPS data. The mobility patterns following the features proposed by Saeb et al [12,13] included location variance, speed mean, speed variance, number of clusters, normalized entropy, homestay, transition time, and total distance. Location variance quantifies the variability in participants’ GPS locations. A higher value of normalized entropy indicates a more uniform distribution of time spent across all locations. Homestay is defined as the proportion of time a participant spends at home. For detailed definitions and formulas of these and other GPS variables, refer to Multimedia Appendix 2.

In this study, active EMA data collection included monitoring participants’ mood, sleep, and exercise states. Participants completed questionnaires every day for 180 consecutive days. The interviewers instructed participants to complete the questionnaire at the times prompted by the app. However, if they missed a prompt, they were allowed to complete it later at a more convenient time. The app prompted participants to complete the mood questionnaire (ie, EMA) twice daily at 12:45 PM and 5 PM, with response rates exceeding 95% for both times. Although participants were encouraged to respond at both times, the responses were generally consistent between the 2 times. Among those who reported twice per day, the intraclass correlations calculated for manic mood=0.888, irritability=0.999, depressed mood=0.920, and fatigue=0.747, indicating low within-day variations between the 2 prompted times. Thus, we selected the 12:45 PM responses for subsequent analysis due to a slightly higher completion rate. The mood questions addressed fatigue, irritability, manic mood, and depressed mood. EMA mood items were adopted from questionnaires that have already been published in papers related to treatment response [23]. They were asked 4 questions related to their current mood: (1) How fatigued do you feel right now? (2) How depressed do you feel right now? (3) How elated do you feel right now? (4) How irritable/angry do you feel right now? Response options included 1 indicating “not at all, I’m in a good mood now,” 2 indicating “a little bit,” 3 indicating “moderate,” 4 indicating “serious,” and 5 indicating “serious and lasted all day.” Participants were given a compensation of approximately US $3.50 each time they completed a month of EMA questionnaires.

### GPS Data Preprocessing and Imputation

**Figure 2:**
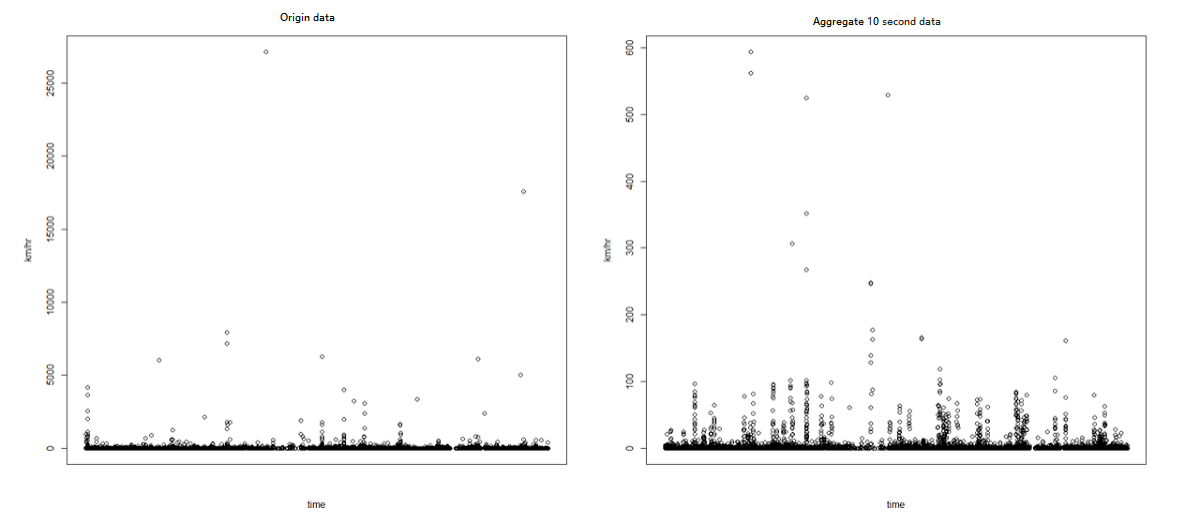
Average speed of the raw data and integrated 10-second data.

Generally, the location coordinate error of GPS is less than 50 m. The raw data are processed following the protocol provided by the Onnela Lab research [22]. The process is as follows:

1. Take data points with an accuracy of <51, indicating high accuracy.
2. Collapse the data points into 10-second intervals. Additionally, calculate the value of longitude and latitude at every 10-second interval, mainly to solve the problem of sudden speed increase caused by GPS drift (Figure 2).
3. Check the distance and time interval between the 2 data points. Moving is a distance that exceeds √10 meters for a 10-second interval. The first stage mainly aimed to confirm (1) moving, (2) pause, (3) cannot define, and (4) missing.
4. The distance between any 2 locations within 300 seconds of <60 m was considered to be a pause at the same location. Represented by the distance length calculated by Mercator projection.
5. Do GPS tracking imputation. Data imputation is prone to achieve real-world distance because the GPS data of smartphones usually contain various degrees of missing data [24].

The following analysis is conducted based on a 10-time simulation average (Multimedia Appendix 3).

### Statistical Analysis

We employed 1-way analysis of variance to analyze differences in the demographics, clinical characteristics, and data collection factors among the heathy controls, BP, and MDD groups. To explore the concurrent relationship between GPS features and EMA mood scores, polyserial correlation was computed. Initially, we analyzed all the samples to identify GPS features that showed a significant correlation with mood variations. The sample size was estimated based on the correlations between GPS features and mood-related symptoms reported in previous literature [12], which ranged between 0.49 and 0.63. Using a correlation coefficient of *r*=0.5, an α level of .05, and desired power levels of .80 and .90, the required sample sizes were 29 and 38, respectively. Thus, the current sample size is sufficient for feature selection in subsequent analyses. Additionally, we applied the generalized estimating equation models to capture the dynamic associations between mobility features and EMA mood states in patients with mood disorders, accounting for time lag associations. This decision was driven by our specific interest in exploring the fluctuations in mood, as captured by the EMA, within the patient group. The working correlation matrix in generalized estimating equation models can also select different types of correlation structures. Typically, the first-order autoregressive model is suitable for studies where the intervals between repeated measurements are of the same length, such as when the time intervals between different time points are identical. For example, in our study, the interval between day 1 and day 2 is one day apart. We considered a 2-sided *P* value of <.05 as statistically significant across all analyses. For the generalized estimating equation models, we adopted the first-order autoregressive working correlation structure to effectively accommodate the within-person correlations over the 6-month duration.

## Results

### Demographic and Clinical Characteristics

During the follow-up period of 6 months, 7 of the 45 participants were lost to follow-up and had <2 weeks of the GPS and EMA data due to various reasons such as the dislike of being located or not being used to filling in questionnaires on a smartphone daily. Therefore, this study analyzed data from 38 participants over a 6-month period, encompassing 5137 person-days. Table 1 demonstrates the sociodemographic characteristics of the participants, including age, gender, social status, as well as the severity of depressive and bipolar symptoms according to affective status. The average age of the participants was 41.34 (SD 12.09) years, with approximately 63% (24/38) of them being female. Additionally, a higher percentage of individuals with BP were unemployed (6/11, 55%) compared to the MDD group and the healthy controls. The adherence rate to the Beiwe app, which calculated the proportion of days on which participants completed EMA mood assessments, was 93.5% (4803/5137) for the entire sample, and the clinical status collection rate was 100%.

**Table 1:** Sociodemographic characteristics of the participants.

	Healthy controls (n=10)	Bipolar disorder (n=11)	Major depressive disorder (n=17)	*P* value
Person-days (n)	1342	1537	2258	N/A^a^
Age (years), mean (SD)	34.40 (11.85)	44.09 (9.97)	43.65 (12.85)	.11
Male gender, n (%)	3 (30)	4 (36)	7 (41)	.86
BDI-II^b^ scores, mean (SD)	4.60 (5.89)	20.73 (12.10)	20.41 (17.29)	.01
HAMD^c^ scores, mean (SD)	—^d^	6.81 (4.79)	7.29 (4.95)	.80
YMRS^e^ scores, mean (SD)	—	5.09 (4.89)	0.53 (1.33)	.001
Unemployment, n (%)	1 (10)	6 (55)	3 (18)	.04
Collection days, mean (SD)	134.20 (73.95)	156.18 (46.72)	135.12 (65.29)	.64
EMA^f^ missing data percentage, mean (SD)	3.48 (3.51)	5.88 (6.29)	8.03 (13.10)	.88

^a^N/A: not applicable.

^b^BDI-II: Beck Depression Inventory-II.

^c^HAMD: Hamilton Depression Rating Scale.

^d^Not available.

^e^YMRS: Young Mania Rating Scale.

^f^EMA: Ecological Momentary Assessment.

### Raw Data Quality and Preprocessing of GPS Data

**Figure 3:**
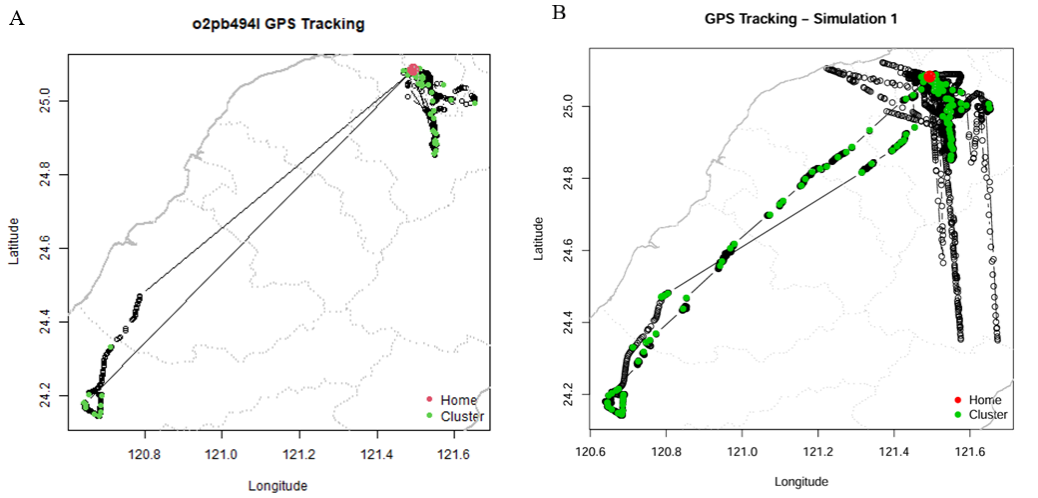
Global positioning system tracking covered on maps. (A) Original track (B) Imputation track. The green circles show the number of clusters. The red circle indicates the location of home. GPS: global positioning system.

We examined both the overall and individual accuracy distributions of the study participants to validate the selection of an accuracy threshold of 50 for the GPS data (Multimedia Appendix 4). Approximately 95% of the overall data points had an accuracy value of <47.59, and approximately 99% had an accuracy value of <76.03. Therefore, setting the threshold at 50 would encompass >90% of the data, with the majority of the accuracy values falling within the range of 10-20 m. This aligns with the recommendations of the research team at the Onnela Lab [24]. The simulated data exhibited a closer resemblance to the moving distance. We present the actual map distribution before and after imputation for 1 participant in Figure 3 to provide visual evidence.

### Two GPS Features Significantly Associated With Depressed Mood in EMA Data

To investigate the potential influences of moods on individual mobility, we conducted a correlation analysis involving GPS features and EMA data for all participants over a 1-week period. The correlation coefficients between 8 GPS features (location variance, speed mean, speed variance, total distance, transition time, number of clusters, homestay, and normalized entropy) and 4 EMA variables (fatigue, depressed mood, manic mood, and irritability) are presented in Table 2, revealing predominantly negative correlations. Notably, the most substantial absolute correlation was observed between depressed mood and location variance (*r*=–0.364; *P*=.03), closely followed by depressed mood and normalized entropy (*r*=–0.353; *P*=.04). Furthermore, it is noteworthy that depressed mood exhibited a positive correlation with homestay (*r*=0.239). Building on these preliminary findings, we then delved into the daily time lag relationship between mood and the aforementioned 3 GPS features by using the 6-month dataset.

**Table 2:** Correlation analysis of the global positioning system features and ecological momentary assessment mood.

	Fatigue		Depressed mood		Manic mood		Irritability	
	Estimate (*r*)	*P* value	Estimate (*r*)	*P* value	Estimate (*r*)	*P* value	Estimate (*r*)	*P* value
Location variance	–0.080	.64	–0.364	.03	–0.098	.57	–0.055	.75
Speed mean	–0.215	.21	–0.186	.28	–0.049	.78	–0.118	.49
Speed variance	–0.194	.26	–0.061	.72	–0.036	.84	–0.173	.31
Total distance	–0.102	.55	–0.305	.07	–0.159	.36	–0.189	.27
Transition time	–0.060	.73	–0.308	.07	–0.177	.30	–0.224	.19
Number of clusters	0.042	.81	–0.139	.42	–0.034	.84	0.090	.60
Homestay	0.142	.41	0.239	.16	0.059	.73	0.058	.74
Normalized entropy	–0.280	.09	–0.353	.04	–0.206	.23	–0.138	.42

### Time Lagging Association of Changes in GPS Features With EMA Mood

We further examined the bidirectional relationship and lagging effect of GPS features on EMA mood by analyzing consecutive 6 months of EMA data and GPS coordinate collection. We evaluated the impact of changes in GPS features on subsequent EMA data as well as the influence of changes in EMA mood on subsequent GPS features. The patient group (Table 3) demonstrated significant associations between changes in GPS features and EMA mood. An increase in location variance from day 1 to day 2 was associated with lower depressed mood levels (β=–0.015; *P*=.009) on day 2. Furthermore, an increase in homestay from day 1 to day 2 was associated with higher fatigue (β=0.256; *P*=.03), depressed mood (β=0.235; *P*=.01), and irritability (β=0.149; *P*=.03) on day 2. However, changes in normalized entropy did not significantly predict any EMA mood within the patient group similar to the overall samples (see Multimedia Appendix 5).

**Table 3:** Time lag association of the change in the global positioning system features with mood for the patient group (bipolar disorder and major depressive disorder).

			Fatigue_day 2_				Depressed mood_day 2_				Manic mood_day 2_				Irritability_day 2_		
			β		*P* value		β		*P* value		β		*P* value		β		*P* value
**Outcome 1**																	
	∆LV^a^_day 1-day 2_	–0.007		.06		–0.015		.009		0.005		.35		–0.004		.31	
	Age	–0.022		.001		–0.017		.04		0.000		.98		–0.003		.62	
	Sex	–0.062		.72		–0.440		.07		–0.367		.04		–0.418		.004	
	LV_day 1_	–0.014		.06		–0.029		.009		0.002		.76		–0.007		.32	
**Outcome 2**																	
	∆NEN^b^_day 1-day 2_	0.206		.31		–0.279		.19		0.065		.58		–0.094		.48	
	Age	–0.023		.001		–0.018		.04		<0.001		.99		–0.003		.60	
	Sex	–0.084		.65		–0.454		.07		–0.372		.04		–0.425		.005	
	NEN_day 1_	0.164		.64		–0.515		.22		0.243		.32		–0.021		.93	
**Outcome 3**																	
	∆HS^c^_day 1-day 2_	0.256		.03		0.235		.01		–0.042		.74		0.149		.03	
	Age	–0.025		<.001		–0.020		.02		<0.001		.99		–0.005		.44	
	Sex	–0.017		.92		–0.419		.08		–0.361		.04		–0.393		.005	
	HS_day 1_	0.460		.002		0.396		.01		0.020		.89		0.249		.07	

^a^LV: location variance.

^b^NEN: normalized entropy.

^c^HS: homestay.

### Time Lagging Association of Changes in EMA Mood With GPS Features

Our analysis of the associations between changes in EMA mood and subsequent GPS features revealed the following results (Table 4). BP and MDD revealed less location variance if fatigue (β=–0.492; *P*=.03) and depressed mood (β=–0.869; *P*<.001) increased from day 1 to day 2. However, the homestay would be greater if fatigue (β=0.047; *P*=.02) and depressed mood (β=0.042; *P*<.001) increased from day 1 to day 2. In all samples (Multimedia Appendix 6), location variance (β=–0.880; *P*=.001) would be less and homestay (β=0.038; *P*=.01) would be greater on day 2 if depressive status increased from day 1 to day 2. Additionally, homestay (β=0.034; *P*=.03) would be higher on day 2 if irritability increased from day 1 to day 2.

**Table 4:** Time lag association of the changes in the ecological momentary assessment mood with global positioning system features for the patient group (bipolar disorder and major depressive disorder).

		Location variance_day 2_		Normalized entropy_day 2_		Homestay_day 2_	
		β	*P* value	β	*P* value	β	*P* value
**Outcome** **1**							
	∆Fatigue_day 1-day 2_	–0.492	.03	0.011	.33	0.047	.02
	Age	0.015	.65	0.000	.79	0.008	<.001
	Sex	1.074	.21	0.038	.33	–0.132	.02
	Fatigue_day 1_	–1.040	.03	0.003	.89	0.097	<.001
**Outcome** **2**							
	∆Depressed mood_day 1-day 2_	–0.869	<.001	–0.014	.21	0.042	<.001
	Age	0.011	.68	0.000	.93	0.007	.002
	Sex	0.378	.64	0.025	.48	–0.108	.07
	Depressed_day__1_	–1.553	<.001	–0.026	.21	0.063	.003
**Outcome** **3**							
	∆Manic mood_day 1-day 2_	0.117	.73	0.008	.38	0.001	.96
	Age	0.039	.26	0.000	.83	0.006	.02
	Sex	1.100	.30	0.045	.28	–0.134	.04
	Manic mood_day 1_	–0.092	.84	0.021	.22	0.013	.76
**Outcome** **4**							
	∆Irritability_day 1-day 2_	–0.498	.15	–0.007	.58	0.040	.01
	Age	0.036	.26	0.000	.83	0.006	.008
	Sex	0.759	.47	0.036	.39	–0.109	.10
	Irritability_day__1_	–0.872	.13	–0.004	.88	0.070	.03

**Figure 4:**
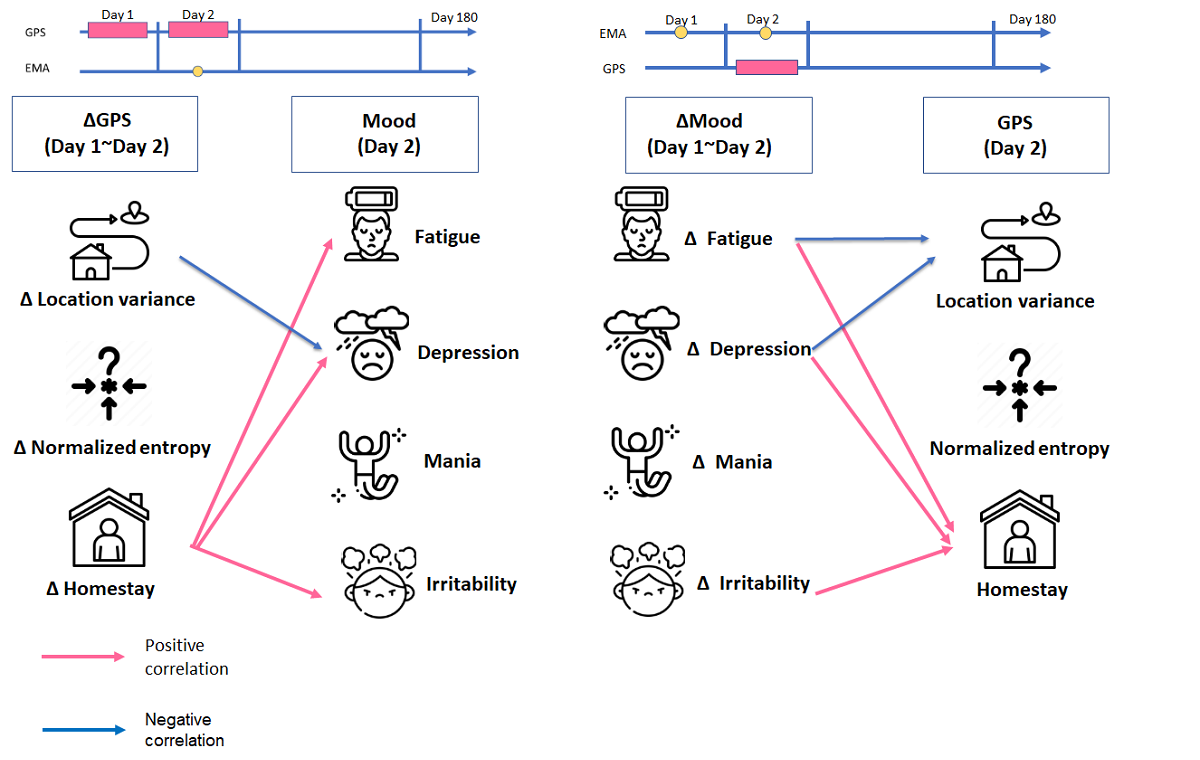
Summary of the time lag effect between the global positioning system features and the ecological momentary assessment moods for bipolar disorder and major depressive disorder. EMA: ecological momentary assessment; GPS: global positioning system.

In our study, we summarized the bidirectional correlations between changes in GPS features and EMA mood assessments in mood disorders, as illustrated in Figure 4. Our key findings are as follows:

1. Significant negative correlations were observed between changes in location variance and depressed mood levels. This suggests that greater variability in location is associated with lower depressed mood scores.
2. We also identified significant positive correlations between increase in homestay duration and elevated levels of fatigue, depressed mood, and irritability. This indicates that spending more time at home is linked to higher levels of these mood symptoms.
3. Additionally, a unidirectional relationship was discovered between increase in fatigue and subsequent changes in location variance. This implies that higher fatigue levels may lead to reduced variability in location.

## Discussion

This study investigates the association between GPS features and EMA mood in individuals with MDD and BP, focusing on correlation analysis and bidirectional time lag effects. Our study enables each participant to generate nearly 100,000 GPS data points by leveraging the abundant data collected from smartphones. GPS-derived mobility patterns are highly individual-specific and can accurately identify individuals based on factors such as their time spent at home, providing a comprehensive representation of their overall statistical mobility patterns [25]. Our study reveals that location variance and normalized entropy, among all GPS features, were moderately correlated with mood symptoms across the entire sample when analyzed on a weekly basis, similar to findings from previous research [12,13,17,26]. Although earlier studies have reported moderate to high correlations (–0.58 to –0.43), our correlations ranging from –0.35 to –0.36 are slightly lower. Additionally, in line with findings from another study [27], mobility patterns correctly classified participants with at least mild depression and those not classified as depressed in 82% of the cases. The most predictive mobility features in that study were related to location changes and entropy, with lower entropy associated with greater inequality in time spent across different locations and higher levels of depression [27]. Furthermore, a study focusing on Android systems revealed correlations among entropy, normalized entropy, location variance, and mood scores [28]. Collectively, our findings indicate that individuals with depressed mood tend to exhibit reduced mobility and a tendency to concentrate in specific locations.

Our study reveals intriguing findings regarding the time lag effect, demonstrating the dynamic bidirectional association between location variance and fatigue and homestay and depressed mood (Figure 4). The bidirectional relationship coincides with previous studies [17] that revealed significant correlations between location variance, homestay, and subsequent depressed mood. Additionally, changes in depressed mood status significantly affected the subsequent periodicity of mobility measured by circadian rhythm. BP and MDD are distinct diagnoses within the mood disorder spectrum, but they share a common feature—the presence of depression. Our study results are consistent with those of a previous study [14] that demonstrated a positive relationship between homestay and the severity of depressive symptoms in individuals with MDD and other affective disorders, including unipolar disorder and BP [29]. Thus, our results support the notion that increased homestay is linked to higher severity of depressed mood. This association suggests that individuals with MDD and BP who spend more time at home may experience decreased energy levels. Feelings of fatigue and loss of energy are relatively easy to observe in the early stages of depression diagnosis [30]. Fatigue, a common early symptom in depression diagnosis, is prevalent in MDD, with over 90% of the patients reporting it as a prodromal or residual symptom [31]. Therefore, prolonged homestay, a key indicator for mood monitoring in our study, is correlated with increased fatigue, depressive symptoms, and irritability in individuals diagnosed with MDD or BP.

Conversely, our findings did not establish a relationship between manic mood states and GPS features. We observed no predictive or time-lagged effects for mania symptoms in BP. This could be attributed to the nature of our study cohort, which, over the 6-month follow-up, predominantly consisted of nonsevere cases due to dropout of more unstable cases. As a result, the low mania scores in the EMA mood assessments were likely due to the lower prevalence of mania symptoms, rendering it challenging to detect any predictors. Additionally, the lack of insight into manic symptoms in BP is a factor that our study could not conclusively address. Although mania is a defining characteristic of BP, depressive episodes and symptoms are more prevalent over the course of the disorder [32]. Thus, even in patients with mania, the response variation to manic states was minimal, leading to more pronounced depressive outcomes in our findings.

Proxies for social and physical behavior, derived from smartphone sensor data such as GPS and phone usage, enable context-sensitive and personalized interventions for individuals with depressive symptoms. Long-term use of the app significantly reduced self-reported symptom severity among participants [33]. In line with this, a study by Rethorst et al [34] explored the efficacy of behavioral activation interventions, which specifically focus on increasing physical activity. Such interventions have shown promise in improving treatment outcomes for depression and enhancing physical health. This underscores the potential benefits of incorporating physical activity into treatment plans for individuals with depression. Additionally, research by Merikangas et al [35] has shown that individuals with bipolar illness exhibit increased cross-domain reactivity, affecting various aspects such as motor activity, sleep, mood, and energy levels. The concept of mobility patterns, akin to activity levels, aligns with the idea that in patients with BP, this heightened cross-domain reactivity suggests a lack of centralized coordination in the network governing activity and energy levels. This is indicative of a broader dysregulation rather than being confined to a single domain. Our study contributes novel insights to this area of research, further elucidating the relationship between activity or mobility levels and mood states. By examining these dynamics in the context of BP, our data provide additional evidence supporting the theory of widespread network dysregulation in these patients, particularly in how their mobility patterns correlate with mood variations.

Previous research has highlighted the predictive power of functional brain connectivity in determining individual mobility patterns. Notably, Xia et al [25] observed that increased connectivity between the somatomotor and limbic networks could predict mobility over medium time scales, ranging from weeks to months. Future research may explore the integration of studies on mobility and brain connectivity, as this could provide valuable insights into the interplay between physical movements and behavioral approaches. Such investigations may deepen our understanding of how brain function influences daily behavior and mobility.

This study has certain limitations that warrant consideration. First, the relatively small sample size poses questions regarding the representativeness of our findings. With a correlation coefficient of 0.364 this study, we achieved a moderate power of .70. A larger scale study is needed to validate these findings. Additionally, it is important to note that despite the limited number of participants, the study capitalizes on the extensive collection of GPS data. These data were gathered daily over a 6-month period, resulting in a substantial volume of person-days EMA and GPS data. Such a dataset offered a more comprehensive view of participants’ behavior over time. Second, data on the Apple iOS version are lacking, which may introduce selection bias. In Taiwan, Apple smartphones hold a market share of 52.55%. Third, the reasons for loss to follow-up varied, with some data missing due to clinical management. When a patient experienced an episode requiring hospitalization during the follow-up period, they were unable to use a mobile phone while hospitalized in Taiwan. Consequently, movement variations during the hospitalization period and EMA completion were not captured, causing a significant amount of missing values due to inpatient health care. In our study, 2 participants lost their data for this reason. Fourth, although participants could complete the questionnaire at convenient times, potentially introducing recall bias, this flexibility likely had minimal impact on our findings. High completion rates and strong intraclass correlations indicate consistent responses across times, suggesting stable mood reporting. Thus, the potential bias from variable response times appears negligible in affecting result reliability. Lastly, the baseline participants demonstrated considerable interest and willingness to answer the app questionnaire daily for 6 months, but a dropout rate of approximately 16% (7/45) was observed at the first 2 weeks during the follow-up.

In conclusion, this study demonstrates a significant correlation between the reported mood states of patients and their mobility patterns. Utilizing a passive method such as carrying a smartphone daily proved to be a convenient and effective way to monitor patients’ conditions. The concept of digital phenotyping employed in this study enabled nonintrusive and noninterfering measurement of mobile behaviors by collecting GPS data from smartphones without causing additional disruptions. Our findings showed a time lag relationship between real-time GPS monitoring combined with EMA. Mobility patterns (location variance and homestay) were significant indicators for detecting poor mood (irritability and depressed mood) and low energy levels (fatigue) in individuals with mood disorders. Therefore, interventions should prioritize promoting physical movement while discouraging prolonged periods of staying at home.

## Appendix

Multimedia Appendix 1 Implementation procedures.

Multimedia Appendix 2 Definition and formula of global positioning system features.

Multimedia Appendix 3 Example of the global positioning system location simulation 10 times.

Multimedia Appendix 4 The distribution of the accuracy value in all the participants (N=38).

Multimedia Appendix 5 Time lag association of the changes in the global positioning system features with the ecological momentary assessment mood for all samples (N=38).

Multimedia Appendix 6 Time lag association of the changes in the ecological momentary assessment mood with the global positioning system features for all the samples (N=38).

## Abbreviations

BP: bipolar disorder
EMA: ecological momentary assessment
GPS: global positioning system
HAMD: Hamilton Depression Rating Scale
MDD: major depressive disorder
YMRS: Young Mania Rating Scale

## Data Availability

The datasets generated and analyzed during this study are not publicly available due to the institutional review board restrictions on direct data sharing. However, they can be made available from the corresponding author upon reasonable request.
