# Supplementary figures and images for: Dynamic Bidirectional Associations Between Global Positioning System Mobility and Ecological Momentary Assessment of Mood Symptoms in Mood Disorders: Prospective Cohort Study
Source: J Med Internet Res. 2024 Dec 6;26:e55635. doi: 10.2196/55635 (PMC11662189; doi:10.2196/55635)

Multimedia Appendix . Implementation procedures.


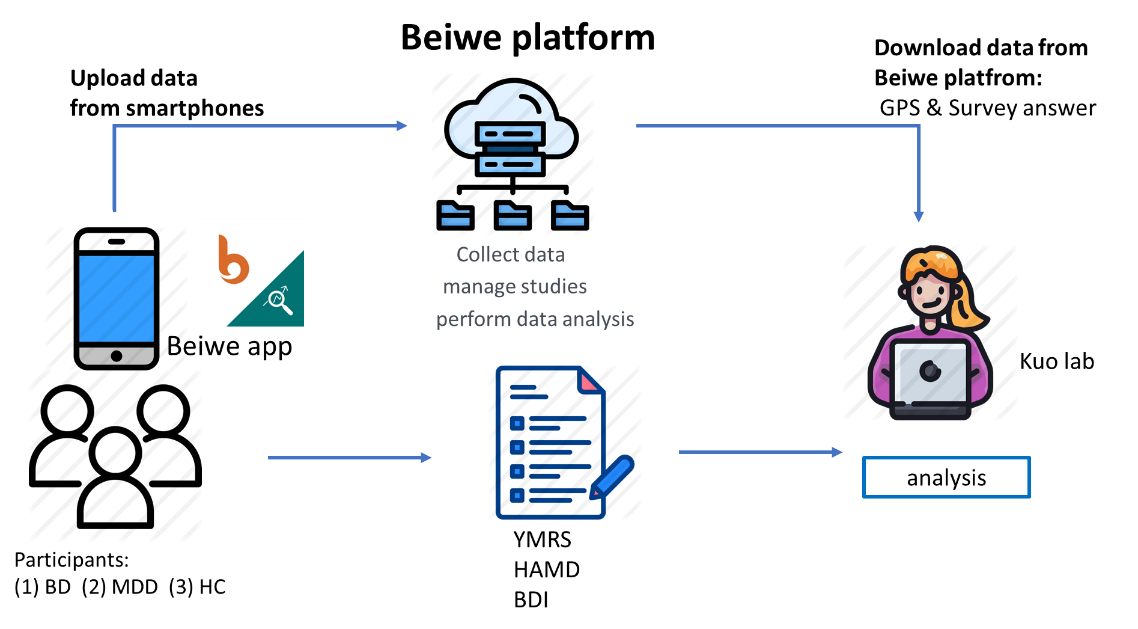

Supplement: Multimedia Appendix 1 [file jmir_v26i1e55635_app1.docx]

Multimedia Appendix . Example for GPS location simulation 10 times.

| 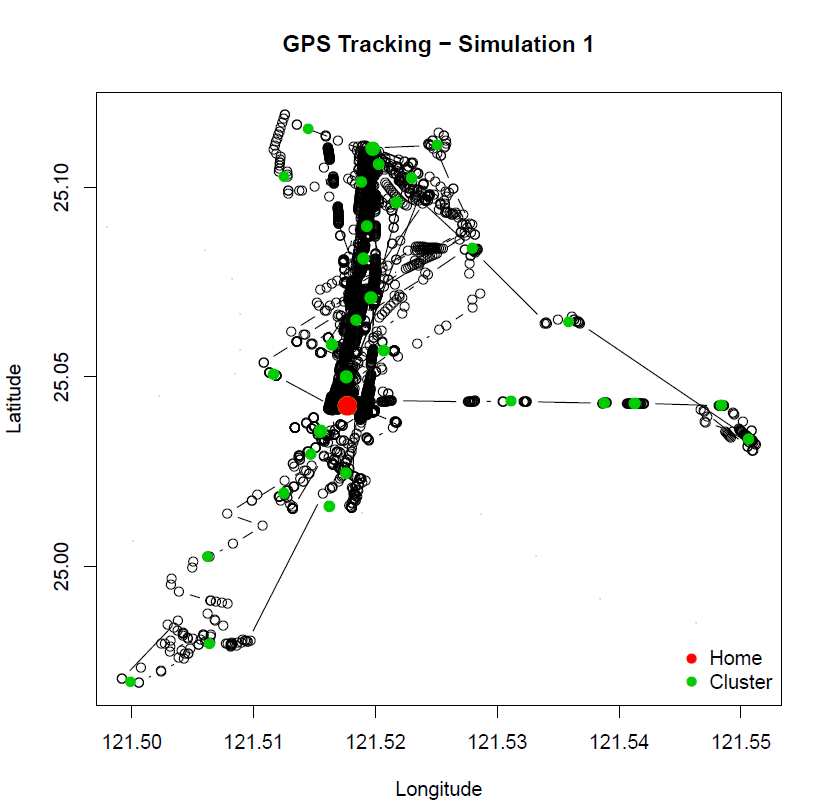 | 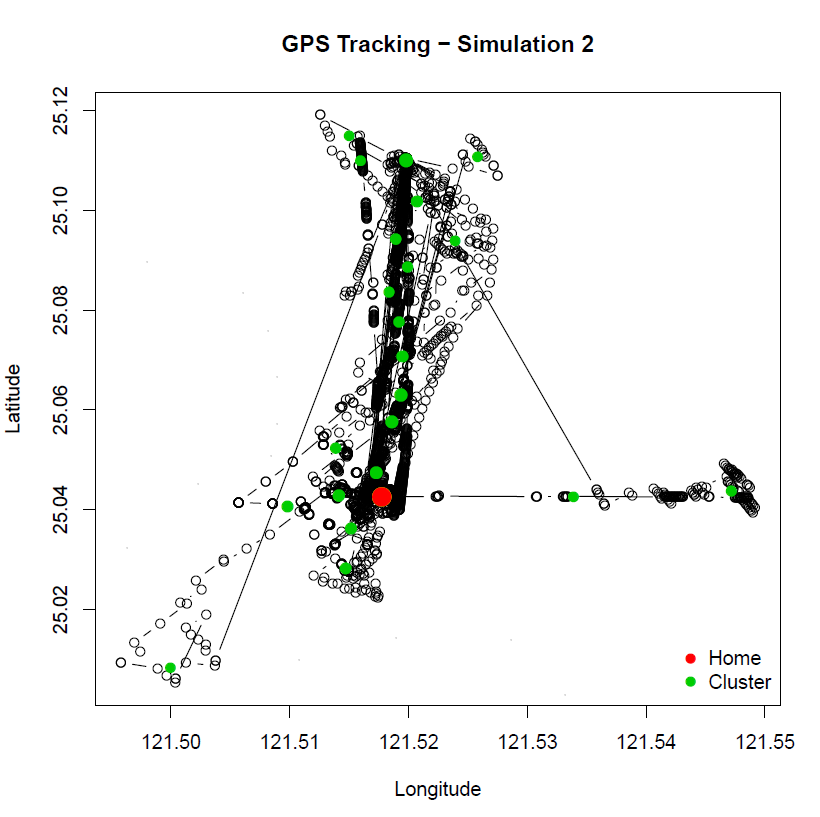 |
| --- | --- |
| 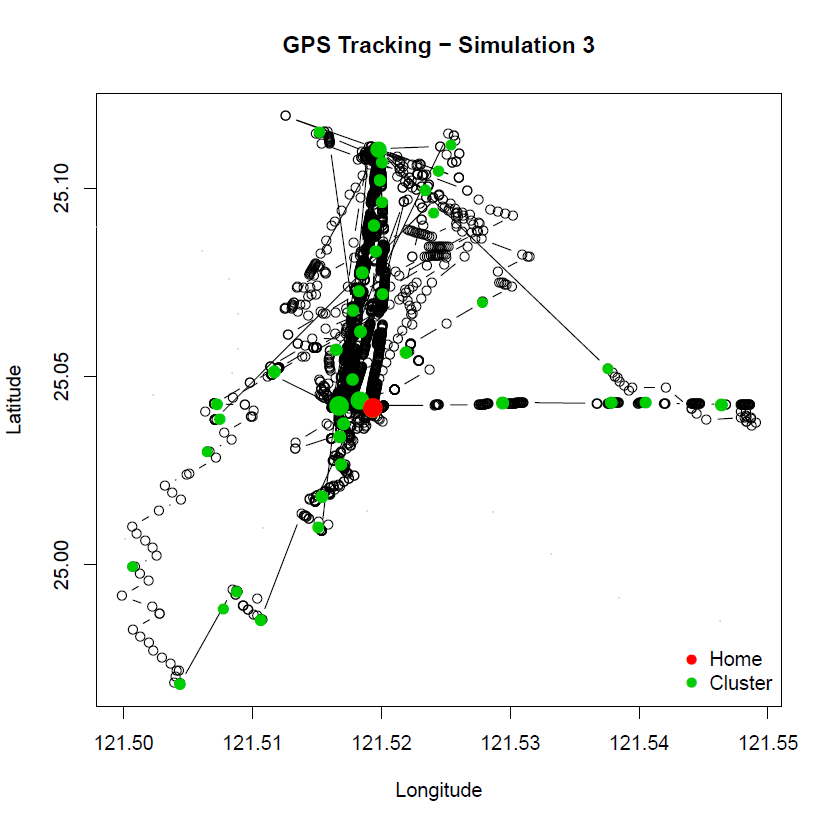 | 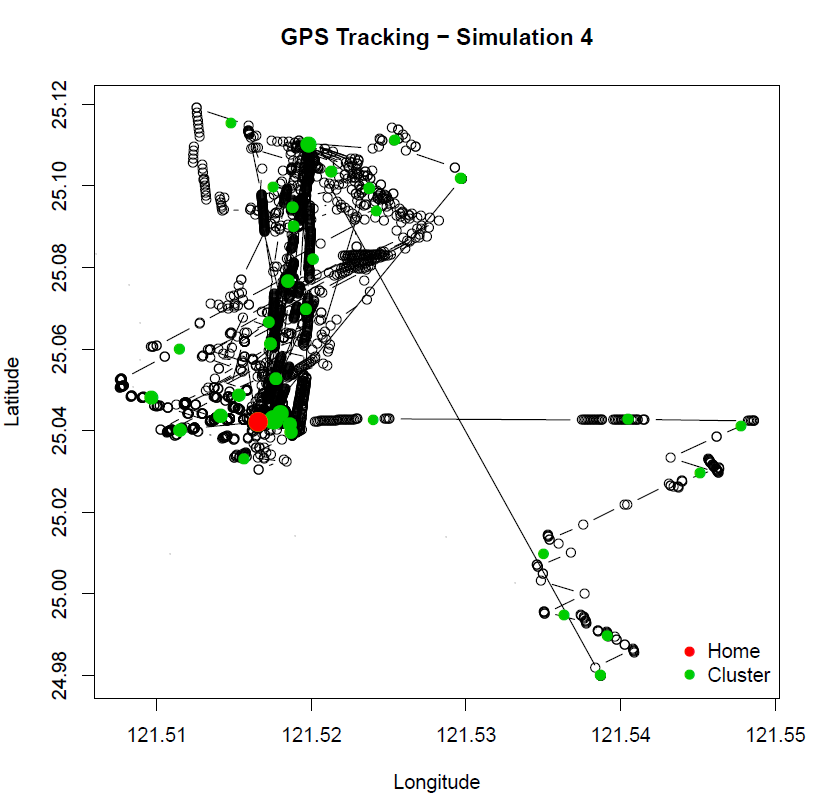 |
| 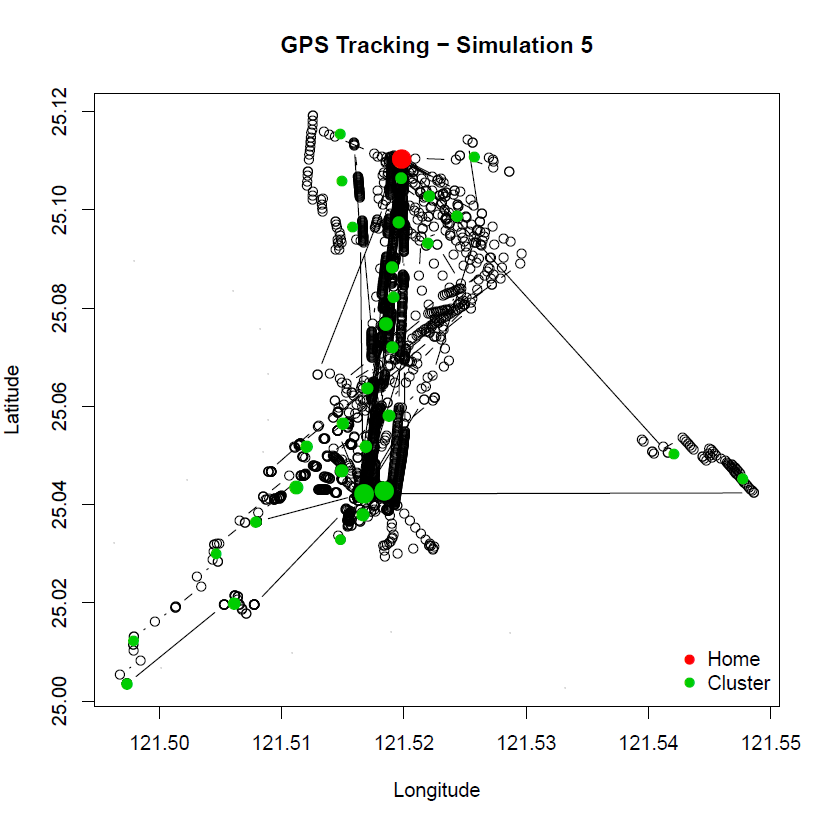 | 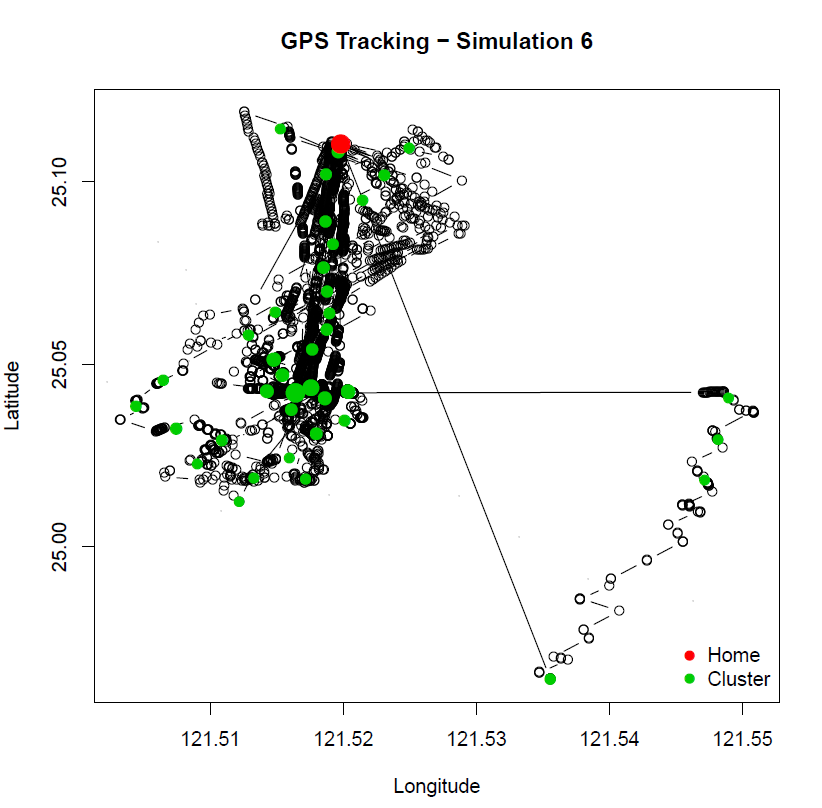 |
| 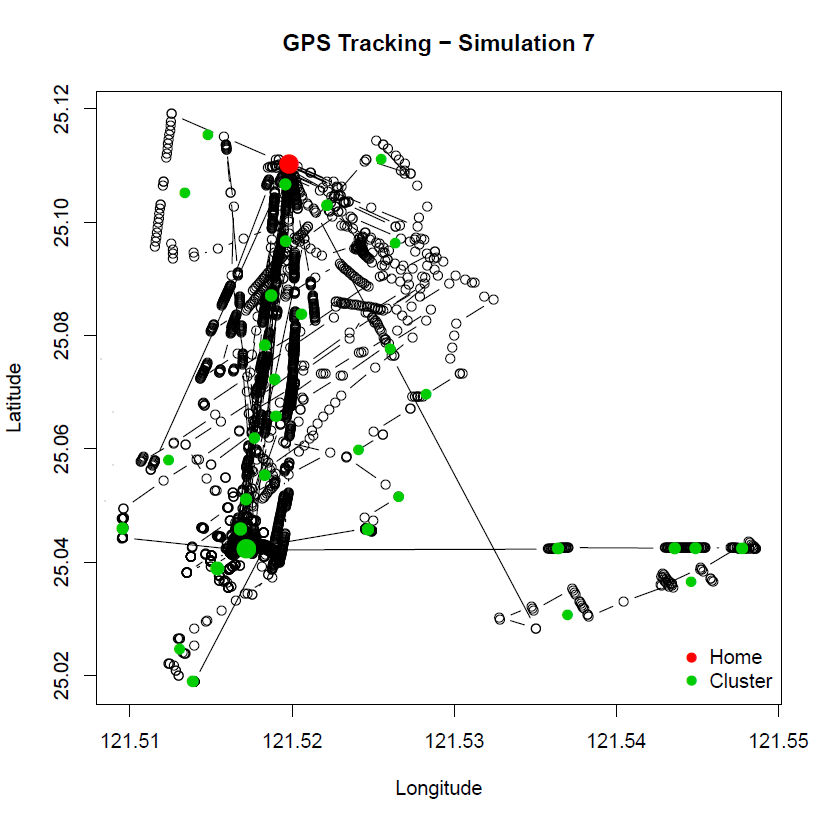 | 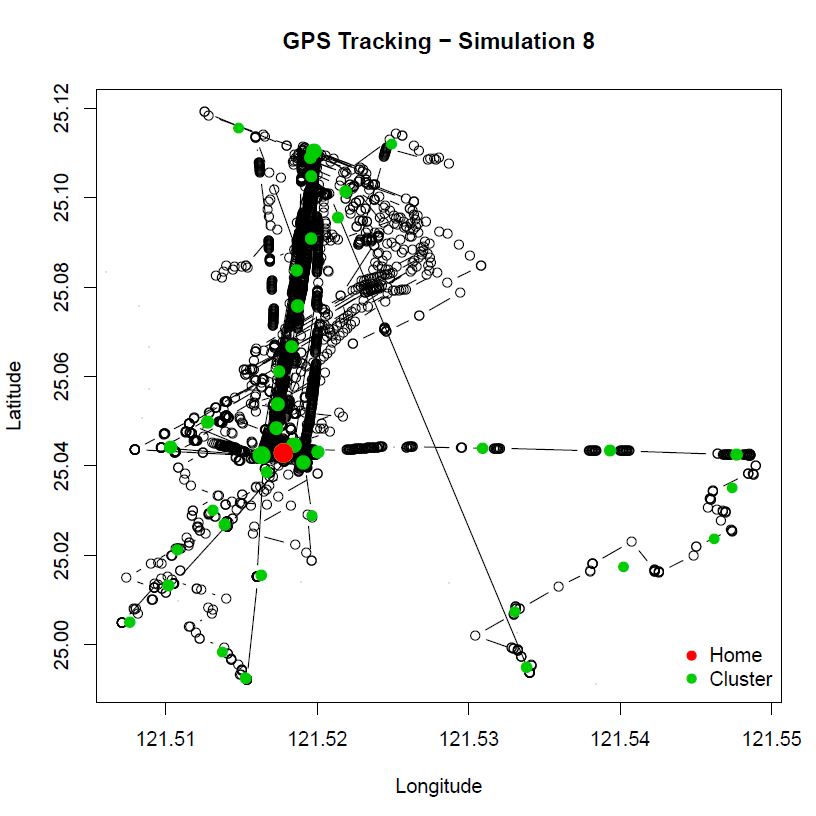 |
| 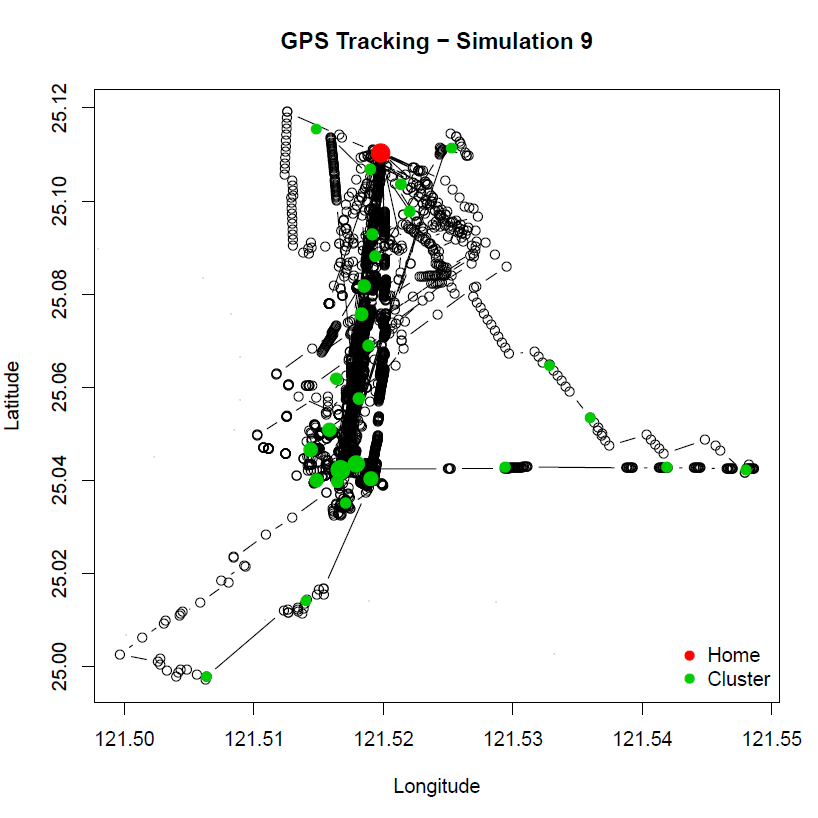 | 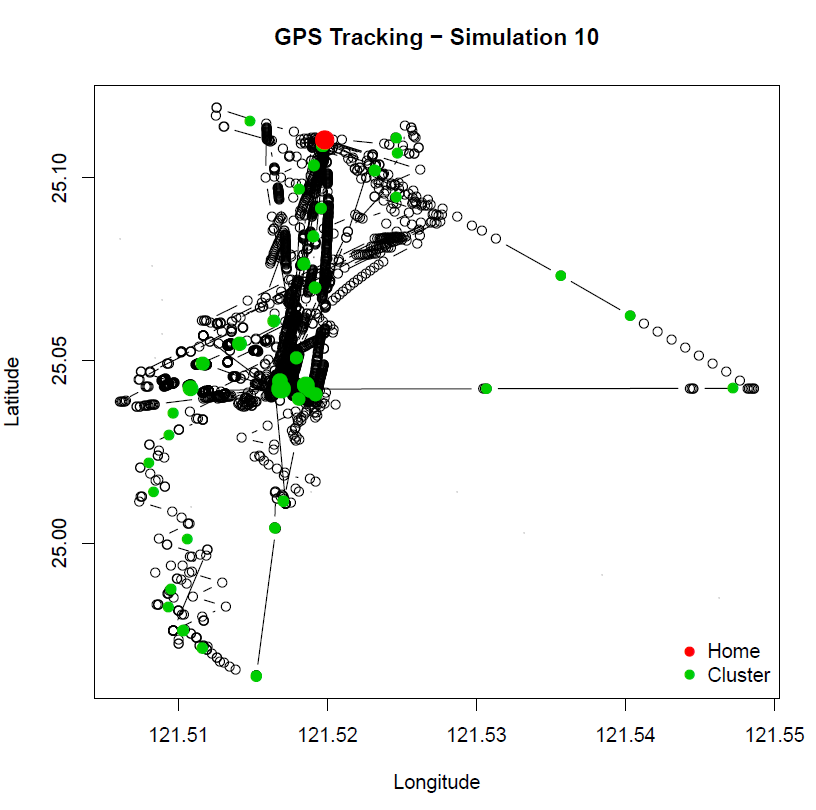 |

Supplement: Multimedia Appendix 3 [file jmir_v26i1e55635_app3.docx]
