# Supplementary material for: Dynamic Bidirectional Associations Between Global Positioning System Mobility and Ecological Momentary Assessment of Mood Symptoms in Mood Disorders: Prospective Cohort Study
Source: J Med Internet Res. 2024 Dec 6;26:e55635. doi: 10.2196/55635 (PMC11662189; doi:10.2196/55635)
Supplement: Multimedia Appendix 2 [file jmir_v26i1e55635_app2.docx]

Multimedia Appendix 2. Definition and formula of GPS features.

| GPS features | definition | formula |
| --- | --- | --- |
| 1. location variance | to measure the variability in a participant’s GPS location | $log\left( \sigma_{lat}^{2}+\sigma_{long}^{2} \right)$ |
| 2. speed mean | Mean of the instantaneous speed obtained at each GPS data point. | $\frac{\Delta\text{distance }}{\Delta time}$ |
| 3. speed variance | Variance of the instantaneous speed |  |
| 4. number of clusters | to identify the places where participants spent most of their time | Number of location clusters found by the adaptive k-means algorithm |
| 5. homestay | Percentage of time spent at home. | 1st-3rd most visited cluster  during the time period between 12 a.m. and 6 a.m |
| 6. transition time | Transition time represented the percentage of time | $\frac{samples in transition states}{total number of samples}$ |
| 7. total distance | The sum of the Euclidean distances between consecutive location points recorded in the data |  |
| 8. normalized entropy | Measured how each participant's time was distributed over different location clusters depends solely on the distribution of the visited location clusters. | Entropy  =-$\sum_{i=1}^{N} pilog(pi)$  *Normalized entropy*  $= \frac{Entropy}{log(N)\text{ }}$ |
