# Supplementary material for: Dynamic Bidirectional Associations Between Global Positioning System Mobility and Ecological Momentary Assessment of Mood Symptoms in Mood Disorders: Prospective Cohort Study
Source: J Med Internet Res. 2024 Dec 6;26:e55635. doi: 10.2196/55635 (PMC11662189; doi:10.2196/55635)
Supplement: Multimedia Appendix 4 [file jmir_v26i1e55635_app4.docx]

Multimedia Appendix 4. The distribution of accuracy value in all participants (N=38).

| A  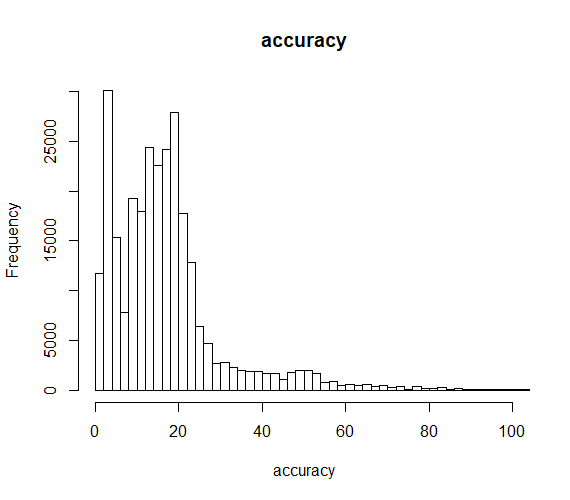 | B  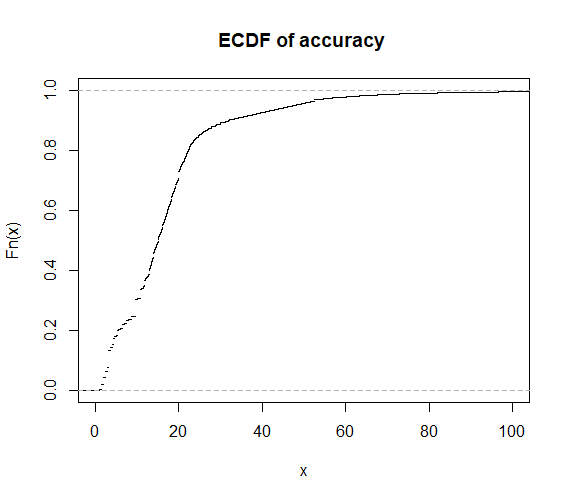 |
| --- | --- |

Figure A shows the frequency corresponding to each accuracy across all participants; Figure B shows the cumulative percentage of all participants below a certain accuracy value (x represents accuracy).
