# Supplementary material for: Dynamic Bidirectional Associations Between Global Positioning System Mobility and Ecological Momentary Assessment of Mood Symptoms in Mood Disorders: Prospective Cohort Study
Source: J Med Internet Res. 2024 Dec 6;26:e55635. doi: 10.2196/55635 (PMC11662189; doi:10.2196/55635)
Supplement: Multimedia Appendix 5 [file jmir_v26i1e55635_app5.docx]

Multimedia Appendix 5. Time-lag association of change in GPS features to EMA mood for all samples (N=38).

| ∆GPS**🡪**EMA mood (all samples) | | | | | | | | | | | |
| --- | --- | --- | --- | --- | --- | --- | --- | --- | --- | --- | --- |
|  | Fatigue _i + 1_ | |  | depression _i + 1_ | |  | mania _i + 1_ | |  | irritability _i + 1_ | |
|  | β | *P* value |  | β | *P* value |  | β | *P* value |  | β | *P* value |
| Outcome1 |  |  |  |  |  |  |  |  |  |  |  |
| ∆LV^a^ _i~i + 1_ | −0.004 | 0.178 |  | −0.012 | 0.018 |  | 0.004 | 0.312 |  | −0.003 | 0.371 |
| BP^b^ | 0.104 | 0.606 |  | 1.005 | 0.000 |  | 0.486 | 0.031 |  | 0.503 | 0.003 |
| MDD^c^ | 0.030 | 0.867 |  | 0.658 | 0.002 |  | 0.189 | 0.030 |  | 0.271 | 0.011 |
| Age | −0.015 | 0.014 |  | −0.015 | 0.011 |  | 0.000 | 0.944 |  | −0.002 | 0.602 |
| Sex | −0.042 | 0.779 |  | −0.322 | 0.099 |  | −0.269 | 0.052 |  | −0.315 | 0.010 |
| LV_i_ | −0.008 | 0.203 |  | −0.021 | 0.027 |  | 0.002 | 0.603 |  | −0.004 | 0.371 |
| Outcome2 |  |  |  |  |  |  |  |  |  |  |  |
| ∆NEN^d^ _i~i + 1_ | −0.043 | 0.791 |  | −0.267 | 0.109 |  | 0.031 | 0.660 |  | −0.096 | 0.256 |
| BP | 0.128 | 0.485 |  | 1.070 | 0.000 |  | 0.473 | 0.032 |  | 0.513 | 0.003 |
| MDD | 0.040 | 0.816 |  | 0.692 | 0.002 |  | 0.184 | 0.034 |  | 0.276 | 0.010 |
| Age | −0.016 | 0.011 |  | −0.017 | 0.006 |  | <0.001 | 0.985 |  | −0.003 | 0.571 |
| Sex | −0.042 | 0.782 |  | −0.326 | 0.100 |  | −0.272 | 0.055 |  | −0.316 | 0.011 |
| NEN_i_ | −0.206 | 0.455 |  | −0.507 | 0.120 |  | 0.144 | 0.329 |  | −0.082 | 0.606 |
| Outcome3 |  |  |  |  |  |  |  |  |  |  |  |
| ∆HS^e^ _i~i + 1_ | 0.132 | 0.205 |  | 0.185 | 0.037 |  | −0.057 | 0.553 |  | 0.103 | 0.034 |
| BP | 0.103 | 0.618 |  | 1.018 | 0.000 |  | 0.486 | 0.032 |  | 0.498 | 0.003 |
| MDD | 0.051 | 0.783 |  | 0.695 | 0.002 |  | 0.185 | 0.036 |  | 0.284 | 0.008 |
| Age | −0.017 | 0.011 |  | −0.017 | 0.007 |  | <0.001 | 0.988 |  | −0.003 | 0.490 |
| Sex | −0.026 | 0.864 |  | −0.316 | 0.109 |  | −0.271 | 0.051 |  | −0.301 | 0.012 |
| HS _i_ | 0.222 | 0.153 |  | 0.268 | 0.084 |  | −0.038 | 0.717 |  | 0.165 | 0.085 |

^a^LV: location variance; ^b^BP: bipolar disorder; ^c^MDD: major depressive disorder; ^d^NEN: normalized entropy; ^e^HS: homestay
