# Supplementary material for: Dynamic Bidirectional Associations Between Global Positioning System Mobility and Ecological Momentary Assessment of Mood Symptoms in Mood Disorders: Prospective Cohort Study
Source: J Med Internet Res. 2024 Dec 6;26:e55635. doi: 10.2196/55635 (PMC11662189; doi:10.2196/55635)
Supplement: Multimedia Appendix 6 [file jmir_v26i1e55635_app6.docx]

Multimedia Appendix 6. Time-lag association of changes in EMA mood to GPS features for all samples (N=38)

| ∆EMA mood 🡪 GPS features (all samples) | | | | | | | | |
| --- | --- | --- | --- | --- | --- | --- | --- | --- |
|  | LV^c^ _i + 1_ | |  | NEN^d^ _i + 1_ | |  | HS^e^ _i + 1_ | |
|  | β | *P* value |  | β | *P* value |  | β | *P* value |
| Outcome1 |  |  |  |  |  |  |  |  |
| ∆fatigue _i~i + 1_ | −0.370 | 0.119 |  | −0.004 | 0.738 |  | 0.027 | 0.192 |
| BP^a^ | −1.062 | 0.198 |  | 0.059 | 0.278 |  | 0.046 | 0.591 |
| MDD^b^ | −0.563 | 0.539 |  | 0.013 | 0.816 |  | −0.067 | 0.392 |
| Age | 0.033 | 0.200 |  | −0.001 | 0.399 |  | 0.005 | 0.017 |
| Sex | 0.974 | 0.179 |  | 0.047 | 0.191 |  | −0.111 | 0.042 |
| fatigue_i_ | −0.809 | 0.093 |  | −0.021 | 0.354 |  | 0.059 | 0.072 |
| Outcome2 |  |  |  |  |  |  |  |  |
| ∆depression _i~i + 1_ | −0.880 | 0.001 |  | −0.019 | 0.072 |  | 0.038 | 0.010 |
| BP | 0.443 | 0.686 |  | 0.094 | 0.133 |  | 0.001 | 0.988 |
| MDD | 0.421 | 0.518 |  | 0.035 | 0.498 |  | −0.099 | 0.171 |
| Age | 0.022 | 0.346 |  | −0.002 | 0.263 |  | 0.005 | 0.015 |
| Sex | 0.455 | 0.482 |  | 0.035 | 0.298 |  | −0.096 | 0.089 |
| depression_i_ | −1.510 | 0.001 |  | −0.035 | 0.077 |  | 0.051 | 0.066 |
| Outcome3 |  |  |  |  |  |  |  |  |
| ∆mania _i~i + 1_ | 0.151 | 0.661 |  | −0.006 | 0.537 |  | −0.006 | 0.828 |
| BP | −1.164 | 0.244 |  | 0.054 | 0.389 |  | 0.054 | 0.517 |
| MDD | −0.608 | 0.506 |  | −0.064 | 0.868 |  | −0.064 | 0.395 |
| Age | 0.047 | 0.088 |  | 0.004 | 0.508 |  | 0.004 | 0.048 |
| Sex | 0.986 | 0.257 |  | −0.114 | 0.186 |  | −0.114 | 0.057 |
| mania_i_ | −0.040 | 0.933 |  | <0.001 | 0.389 |  | <0.001 | 0.999 |
| Outcome4 |  |  |  |  |  |  |  |  |
| ∆irritability _i~i + 1_ | −0.443 | 0.166 |  | −0.010 | 0.369 |  | 0.034 | 0.033 |
| BP | −0.770 | 0.417 |  | 0.062 | 0.291 |  | 0.027 | 0.750 |
| MDD | −0.386 | 0.677 |  | 0.015 | 0.791 |  | −0.079 | 0.292 |
| Age | 0.045 | 0.081 |  | −0.001 | 0.484 |  | 0.004 | 0.033 |
| Sex | 0.741 | 0.389 |  | 0.044 | 0.257 |  | −0.097 | 0.111 |
| irritability_i_ | −0.774 | 0.131 |  | −0.010 | 0.661 |  | 0.054 | 0.089 |

^a^BP: bipolar disorder; ^b^MDD: major depressive disorder; ^c^LV: location variance; ^d^NEN: normalized entropy; ^e^HS: homestay
